# Longitudinal Study of Three microRNAs in Duchenne Muscular Dystrophy and Becker Muscular Dystrophy

**DOI:** 10.3389/fneur.2020.00304

**Published:** 2020-04-21

**Authors:** Selena Trifunov, Daniel Natera-de Benito, Jesica Maria Exposito Escudero, Carlos Ortez, Julita Medina, Daniel Cuadras, Carmen Badosa, Laura Carrera, Andres Nascimento, Cecilia Jimenez-Mallebrera

**Affiliations:** ^1^Neuromuscular Unit, Neuropaediatrics Department, Institut de Recerca Sant Joan de Déu, Hospital Sant Joan de Déu, Barcelona, Spain; ^2^Rehabilitation and Physical Unit Department, Hospital Sant Joan de Deu, Barcelona, Spain; ^3^Statistics Unit, Fundación Sant Joan de Déu, Barcelona, Spain; ^4^Biomedical Network Research Centre on Rare Diseases (CIBERER), Instituto de Salud Carlos III, Madrid, Spain

**Keywords:** microRNAs (miRs), Duchenne muscular dystrophy (DMD), Becker muscular dystrophy (BMD), biomarker, long-term follow up

## Abstract

Our objective was to investigate the potential of three microRNAs, miR-181a-5p, miR-30c-5p, and miR-206 as prognostic biomarkers for long-term follow up of Duchenne muscular dystrophy (DMD) and Becker muscular dystrophy (BMD) patients. We analyzed the expression of three microRNAs in serum of 18 patients (DMD 13, BMD 5) and 13 controls using droplet digital PCR. Over 4 years a minimum of two and a maximum of three measurements were performed at different time points in the same patient. Correlations between microRNA serum levels, age, and functional outcome measures were analyzed. We show the individual evolution of the levels of the three microRNAs in 12 patients and also the effect of corticosteroid treatment on microRNAs expression. We measure the expression of three microRNAs in the muscle of six DMD patients and also the expression of target genes for miR-30c. We found that levels of miR-30c and miR-206 remained significantly elevated in DMD patients relative to controls over the entire study length. The introduction of the corticosteroid treatment did not significantly influence the levels of these microRNAs. We report a trend for microRNA levels to decrease with age. Moreover, miR-206 expression levels are capable to distinguish DMD from BMD patients according to ROC analysis. We found miR-30c expression decreased in the muscle of DMD patients and marked upregulation of the target genes for this microRNA. MiR-30c and miR-206 represent sensitive biomarkers for DMD, while miR-206 may have an additional value to distinguish the DMD and BMD phenotype. This may be particularly relevant to assess the effectiveness of treatments aimed at converting the DMD to the less-severe BMD like phenotype.

## Introduction

Muscular dystrophies represent a heterogeneous group of disorders both from the genetic and phenotypic point of view, with muscle wasting as a common trait. Duchenne muscular dystrophy (DMD) is the most common childhood muscular dystrophy, affecting one in 5,000 male births and is caused by mutations in the X-linked DMD gene resulting in the absence or severe reduction of the dystrophin protein. A milder phenotype of Becker muscular dystrophy (BMD) arises from in-frame mutations in the DMD gene resulting in a production of truncated, partially functional protein (1). The current genetic prediction of whether a mutation will result in a DMD or BMD phenotype is the reading-frame rule (Monaco rule) (2). By this rule, the maintaining of an open reading frame (ORF) in the spliced mRNA despite a deletion event would give rise to the BMD phenotype while not maintaining an ORF would give rise to the DMD phenotype. The reading-frame rule is applicable for the vast majority of patients; however, numerous exceptions of the rule also exist (3). The mutational variability seen in DMD and BMD patients is normal if the enormous size of the DMD gene is taken into account (4). Consequently, there is a wide clinical spectrum of severity that does not always allow patients to be categorized as DMD or BMD, especially at early stages of the disease (5). This is particularly important for adequately selecting patients in clinical trials, when a clear phenotype is required (6). Thus, in addition to the biomarkers for the early diagnosis and to aid more accurate patient phenotype classification. There is also a need for monitoring biomarkers as patients clinical responses to gene therapy treatment are heterogeneous (7). This would lead to more specific clinical management and allow more personalized treatments for patients in the future. Moreover, new treatment strategies require a measurable outcome of patient responses (8). Thus there is an unmet need for the predictive/biomarkers. Significant efforts are being made by researchers in the field to identify valuable biomarkers in easily accessible tissues, such as serum. microRNAs (miRNAs) are one class of biomarkers that have drawn much attention in the DMD biomarker field. MiRNAs are non-coding RNAs, 19–22 nucleotides long, which act as post-transcriptional regulators of gene expression. The role of miRNAs in the regulation of key biological processes in skeletal muscle is well-established (9) and they serve as diagnostic, prognostic biomarkers, and as molecular outcome measures in various biomedical fields (10, 11). Previously, our group used gene network analysis to identify two miRNAs, miR-30c, and miR-181a and validated them as reliable serum diagnostic biomarkers for DMD (12). Also, there is a group of miRNAs called dystromirs (miR-1, miR-133a, miR-133b, miR-31, and miR-206) that are muscle-specific and elevated in the serum of DMD patients (13). In the context of DMD may be the most interesting dystromir is miR-206 which mediates the increase of utrophin (utrophin is paralogous to dystrophin) expression in skeletal muscles, thus may serve as a potential therapeutic target for DMD. MiR-206 is primarily expressed in newly formed myotubes or regenerated fibers as it plays an essential role in the pathological process of skeletal muscle injury and regeneration (14). The expression of miR-206 is elevated both in mdx mice and in patients with DMD (15). Ambulant patients have a higher level of miR-206 and other dystromirs compared to non-ambulant patients, which can be attributed to pathological progression and/or higher levels of physical activity. It was found that miR206 levels increase with age in younger patients with DMD (age 2–6 years) (16). This can be explained by the fact that DMD patients undergo a period of normal childhood growth that may compensate for myofiber degeneration (17).

These previously validated miRNAs can differentiate DMD patients from controls with BMD patients having intermediate levels of these miRNAs to levels seen in DMD patients and controls (8). However, there is little information regarding changes in miRNAs abundances/expression over time. Previous studies used age as an indicator of disease progression and compare miRNA levels in patients of different ages (18). Also, the effects of corticoid treatment are most commonly accessed between the treated and untreated groups of patients. However, a longitudinal analysis in the same patients would give a more wholesome insight into the ability of the dystromirs to represent individual disease trajectories and disclose the effect of corticoid treatment on an individual level.

Here, we assessed miR-30c, miR181-a, and miR-206 concentrations in serum samples of DMD and BMD patients over 4 years. To determine their potential use as surrogate markers of disease severity we looked for correlations between miRNA serum concentrations and functional scores and compared DMD vs. BMD patients.

Besides, to answer if age and ambulatory status influence the biomarker capacity of miR-30c and miR-206 we examined a wider cohort of patients. We observe that the levels of all three miRNAs investigated decrease with the age of the patients, but a statistically significant decrease is observed only for miR-206. We also do not observe significant changes in levels of miRNAs in patients after the introduction of the corticoid treatment. We find that miR-206 serum concentrations can discriminate between DMD and BMD patients at any stage of the disease.

## Materials and Methods

### Ethics Statement

This work has been approved by the Ethical Committee of “Fundació Sant Joan de Déu.” Written informed consent for research was obtained from all patients and controls (or their parents/legal guardians) according to the Hospital Sant Joan de Déu forms and regulations.

#### Study Participants

Longitudinal study participants: We included 18 patients (Table 1) for which at least two different time-point measurements were performed (DMD 13, BMD 5). For 12 of those patients (DMD 7 and BMD 5) three different time point measurements were obtained.

**Table 1:** Longitudinal study participants.

	**First measurement**		**Second measurement**		**Third measurement**	
	***n***	**Median age and age range**	***n***	**Median age and age range**	***n***	**Median age and age range**
DMD	10	7.5 years, range 3–13 years	11	8.7 years, range 4–14 years	16	10.2 years, range 6–16 years
BMD	5	12.6 years, range 9–15 years	5	13.6 years, range 10–16 years	5	15.6 years, range 13–18 years

Not longitudinal study participants: We recruited an additional cohort of very young DMD patients (*n* = 5, median age = 3.5 years, range 2–5 years), not ambulant DMD patients (*n* = 6, median age = 14.4 years, range 12–17 years), and BMD patients (*n* = 10, median age = 21.3 years, range 2–65 years) including two families where we have analyzed miRNA levels in more than one affected family member.

### Outcome Measures

The following functional outcome measures were performed at each time point in ambulant DMD and BMD patients. These tests provide a measure of physical ability in ambulatory DMD patients. Six-Minute Walk Test (6-MWT) measures the distance a patient can walk in 6 min on a hard, flat surface, according to the ATS guidelines. NSAA is a scale consisting of 17 items, ranging from standing (item 1) to running (item 17) and including several abilities such as head raise, hopping, or standing on heels. Each item is scored on a 3-point scale using the following criteria: 2, Normal achieves the goal without any assistance; 1, Modified method but achieves goal independent of physical assistance from another person; 0, Unable to achieve independently. A total score can be obtained by summing the scores for all the individual items. The score can range from 0 (absence of ambulation) to 34 (normal ambulation) Timed Function Tests (TFTs) included time taken to rise from floor, run/walk 10 m, climb 4 standard-sized stairs, and descend 4 standard-sized stairs.

### Blood Samples, RNA Isolation, and Quantification for Reverse Transcription and ddPCR

Peripheral venous blood was collected first thing in the morning before clinical examination and functional assessment at Hospital Sant Joan de Déu in serum tubes (Vacuette), kept at room temperature to clot for 30 min in vertical position, and then spun at room temperature at 3,000 rpm for 10 min; the serum was removed and dispensed in aliquots. No signs of hemolysis were detected. Aliquots were stored at −80°C until use. Total RNA including miRNAs was extracted from 200 μL of serum using TRIzol/Chloroform (Life Technologies) according to the protocol previously described. Briefly, after the sample was mixed with 1 mL of TRIzol, 3 μL of 5 nM synthetic spike-in cel-miR-39-3p from Caenorhabditis Elegans (custom synthesized by Integrated DNA Technologies) was added. Before precipitation of RNA with isopropanol, 1 μL of RNase-free glycogen (Life Technologies) was added to improve extraction efficiency. RNA was eluted in 30 μL of nuclease-free water. For this study, we used fluorescent quantification of miRNAs with the Qubit system (Life Technologies, catalog number: Q3288), following manufacturer's instructions.

Three circulating miRNAs (miR-181a-5p, miR-30c-5p, and miR-206) were reverse-transcribed individually from human serum samples using TaqMan miRNA Reverse Transcription kit (Life Technologies, cat no. 4366596) and miRNA-specific stem-loop primers (Life Technologies). For each sample, 3.34 μL of RNA was reverse transcribed in a 10 μL reaction using the standard protocol and primers specific for the three miRNAs: miR-181a-5p (assay ID: 00480), miR-30c-5p (assay ID: 000419). The amount of primers and cDNA for each assay as well as the annealing temperature for PCR amplification were optimized. Then, 1 μL of the resulting cDNA (undiluted for miR-181a and miR-206 or diluted 1:5 for miR-30c) was prepared for amplification in a 20 μL reaction volume containing 10 μL of 2X ddPCR Supermix for Probes (Bio-Rad), 1 μL of 20X TaqMan miRNA PCR primer probe set, and 7.67 μL of nuclease free water. ddPCR assay mix (20 μL) was loaded into the wells of a disposable DG8 cartridge (Bio-Rad) with 70 μL of droplet generation oil for probes (Bio-Rad). The cartridge was then placed into the QX200 Droplet Generator (Bio-Rad). Between 10,000 and 20,000 highly uniform nanoliter-sized droplets were generated in each well and transferred to a 96-well PCR plate (Eppendorf, Germany) and the plate was sealed. PCR amplification was performed in a thermal cycler (Bio-Rad) at 95°C for 10 min, then 40 cycles of 94°C for 30 s and 56°C for 1 min (ramping rate reduced to 2%), and a final inactivation step at 98°C for 10 min. After PCR, the plate was loaded into the QX200 Droplet Reader (Bio-Rad) for automatic reading of positive (did contain target) and negative (did not contain target) droplets in each sample. All samples were run in duplicate and a no-template control (NTC) was included in every assay. The data were analyzed using the QuantaSoft software™ (Bio-Rad). Briefly, discrimination between negative and positive droplets was achieved by setting manually a fluorescence amplitude threshold for each microRNA assay based on results from NTC wells. The absolute amount of each microRNA was calculated by counting the number of positive droplets per panel. The corrected number of targets (determined by Poisson statistical analysis) was multiplied by the corresponding dilution factor to obtain the total copy number per μL of PCR mixture.

#### Normalization of Copies of the Target cDNA for Different DNA Concentrations

Taking into account that the initial concentration of miRNAs is different across the samples, we have applied the normalization of the number of copies/μL to express all the ddPCR results in copies/ng.

### Statistical Analysis

Statistical analysis was performed using R 3.0.2 and PRISM 8.0 software. A non-parametric test, Mann–Whitney sum test, was used to compare miR-30c, miR-181a, and miR-206 expression levels between two groups. Spearman's rank correlation was applied to correlate miR-30c, miR-181a, and miR-206 expression levels with age and functional outcome measurements. The receiver-operator characteristic (ROC) curve and area under the curve (AUC) analyses were applied to determine the diagnostic accuracy of each microRNA. A *p* ≤ 0.05 was considered significant. Repeated measures ANOVA was used to compare the evolution of the biomarkers between the two groups.

## Results

### Biomarker Expression Levels in DMD and BMD Patients Throughout Longitudinal Study

**Figure 1:**
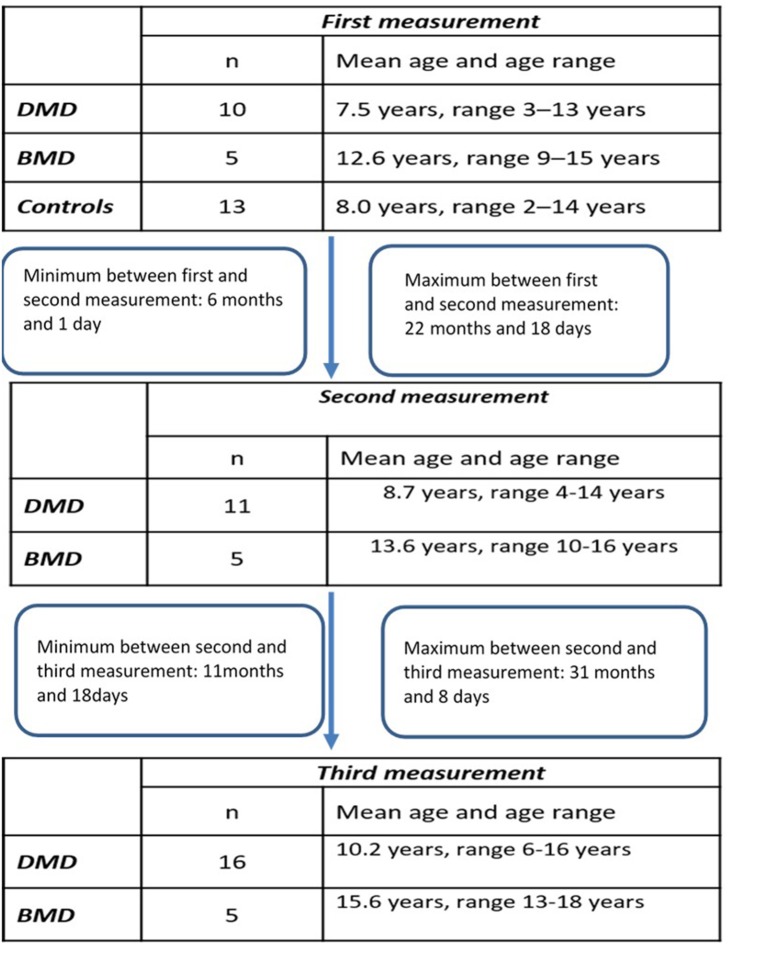
Flowchart of the study.

The design of the study has been illustrated in the Flowchart 1 (Figure 1).

The expression levels are determined in control population for miR-181a (*n* = 13, mean = 9.753 copies/ng), miR-30c (*n* = 13, mean = 60.35 copies/ng), and mir-206 (*n* = 13, median = 1.025 copies/ng) and compared to the expression levels measured in the DMD and BMD patients at different time points.

#### Longitudinal Analysis of miR-30c

**Figure 2:**
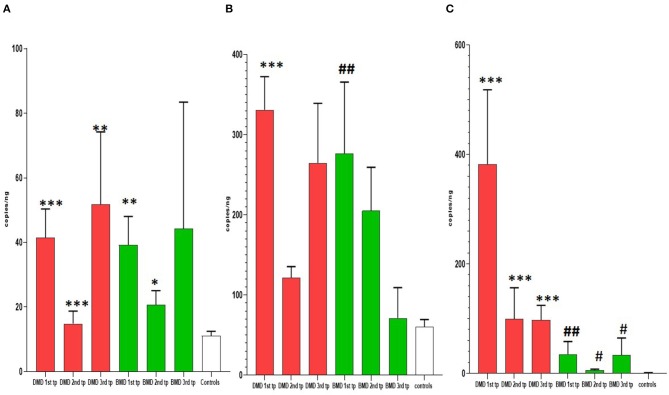
Results of longitudinal analysis of miRNAs expression. The *X*-axis shows three different time points (first, second, and third) of measurements in DMD and BMD patients; *Y*-axis shows the number of copies per nanogram. The borders of the columns represent mean values and error bars present SEM. For all time points, three independent measurements are performed in duplicates. **(A)** Longitudinal expression of miR-30c, **(B)** longitudinal expression of miR-181a, **(C)** longitudinal expression of miR-206. Levels of significance for comparison of DMD patients to controls are noted as *, **, ***; for comparison of BMD to controls #, ##, ###. *P < 0.05; **P = 0.001−0.01; ***P < 0.001. #*P* < 0.05; ##*P* = 0.001−0.01.

In the first time point miR-30c levels were significantly higher (*p* < 0.0001) in DMD patients (mean = 330.9 copies/ng) and in BMD patients (*p* = 0. 0016, mean = 276.6 copies/ng) relative to control. This differences were maintained in the second time point for DMD patients (mean = 121.7 copies/ng) and BMD patients (mean = 205.2 copies/ng), *p* = 0.0048 and 0.0350, respectively. In the third measurement in DMD patients the levels of miR-30c were significantly higher (*p* = 0.0087, mean = 264.8 copies/ng) but the significance was lost for the BMD group (mean value = 71.25 copies/ng; Table 2, Figure 2).

**Table 2:** Descriptive statistics of miR-30c expression in DMD and BMD patients.

	**miR-30c**						
	**First-time point**		**Second-time point**		**Third-time point**		
	**DMD**	**BMD**	**DMD**	**BMD**	**DMD**	**BMD**	**Controls**
*N*	10	5	11	5	16	5	13
Min	171.5	86.5	45.74	40.58	19.34	9.033	21.99
Max	634.8	581.9	195.7	331.3	1,028	202.5	142.8
Range	463.3	495.4	150	290.7	1,008	193.5	120.8
Mean	330.9	276.6	121.7	205.2	264.8	71.25	60.35
SD	131.7	199.3	45.72	121.1	297.7	85.19	32.89
SEM	41.65	89.13	13.78	54.16	74.42	38.1	9.121

#### Longitudinal Analysis of miR-181a

In the first time point miR-181a levels were significantly higher (*p* < 0.0001) in DMD patients (mean value = 41.53 copies/ng) and in BMD patients (mean value = 39.24 copies/ng) with (*p* = 0.0016) in respect to controls. However, in the second and third time point we did not observe significant differences in DMD (mean = 14.83 and 51.75 copies/ng) and BMD patients (means = 20.69 and 44.24 copies/ng; Table 3, Figure 2).

**Table 3:** Descriptive statistics of miR-181a expression in DMD and BMD patients.

	**miR-181a**						
	**First-time point**		**Second-time point**		**Third-time point**		
	**DMD**	**BMD**	**DMD**	**BMD**	**DMD**	**BMD**	**Controls**
*N*	10	5	11	5	16	5	13
Min	15.26	17.44	1.33	5.84	0.85	1.55	5.61
Max	114.2	69.83	44.68	30.91	358	201	21.03
Range	98.95	52.39	43.35	25.07	357.2	199.4	15.42
Mean	41.53	39.24	14.83	20.69	51.75	44.24	11.1
SD	27.93	19.66	12.97	9.781	89.92	87.73	5.033
SEM	8.832	8.792	3.911	4.374	22.48	39.23	1.396

#### Longitudinal Analysis of miR-206

In the first time point miR 206 levels were significantly higher (*p* < 0.0001) in DMD patients (mean value = 382.3 copies/ng) and in BMD patients (mean value = 35.10 copies/ng) in respect to controls. Also in the second time point DMD patients (mean value = 99.29 copies/ng) and BMD patients (mean value = 5.896 copies/ng) have significantly higher values (*p* < 0.0001 and *p* = 0.021, respectively) of miR 206. In the third measurement in DMD patients (*n* = 16, mean value = 97.33 copies/ng) the levels of miR206 were significantly higher (*p* < 0. 0001) then in controls. The significance (*p* = 0.027) was also found for the BMD patients (mean value = 33.65 copies/ng) in the third year time point (Table 4, Figure 2).

**Table 4:** Descriptive statistics of miR-206 expression in DMD and BMD patients.

	**miR-206**						
	**First-time point**		**Second-time point**		**Third-time point**		
	**DMD**	**BMD**	**DMD**	**BMD**	**DMD**	**BMD**	**Controls**
*N*	10	5	11	5	16	5	13
Min	9.63	1.963	4.977	0.5509	2.342	1.108	0.1912
Max	1,300	126	662.9	12.24	336.0	158.0	3.135
Range	1,290	124	657.9	11.69	333.7	156.9	2.944
Mean	382.3	35.1	99.29	5.896	97.33	33.65	1.338
SD	429	52	189.4	4.297	108.7	69.52	0.9518
SEM	135.7	23.25	57.11	1.922	27.18	31.09	0.264

#### Comparison of the miRNA Signature in DMD vs. BMD

**Figure 3:**
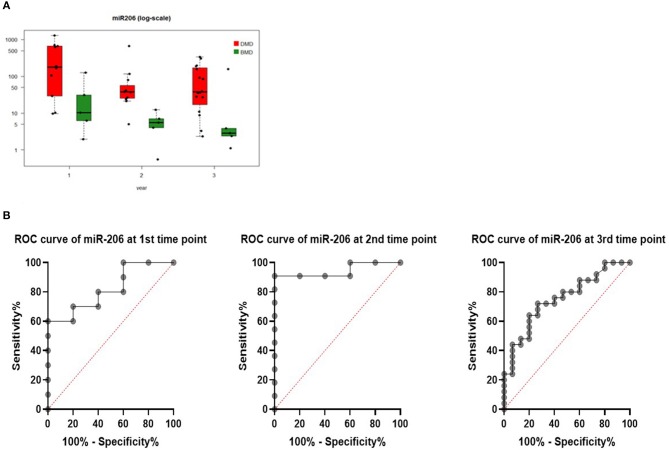
**(A)** Boxplot of expression of miR-206 in DMD and BMD patients shown in three different time points (1, 2, and 3) on the *X*-axis. *Y*-axis shows the number of copies per nanogram. Lines in the box represent median values, the borders of the box are first and third quartile. The whiskers present the minimum and maximum. Separate dots represent outliers. **(B)** ROC curves for miR-206 at first, second, and third-time point measurements.

Levels of miR206 showed significant differences between DMD and BMD patients throughout the entire study (*p* < 0.05; Figure 3A). The biggest difference was observed at the start of the study (~100 times higher in DMD than BMD) whilst this decreased to 6-fold at the last time point.

To evaluate if serum levels of miR-206 can be used as potential marker to separate DMD from BMD, ROC curve analyses were performed for each time point and they have shown that miR-206 levels were able to discriminate DMD from BMD patients with AUC of 0.82, 0.95, and 0.75 in first, second, and third-time point, respectively (Figure 3B).

**Figure 4:**
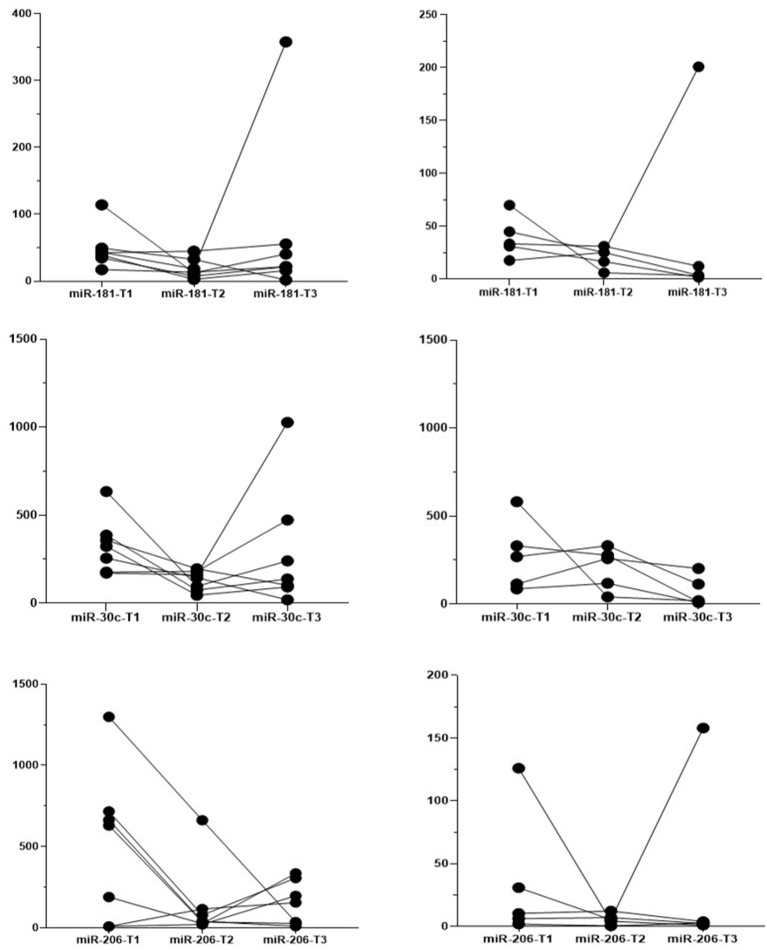
Individual evolution of the biomarkers. Each line presents one patient, the dots present the measured expression levels at the time point. Left-hand side graphs represent DMD patients (*n* = 7) and the right-hand side BMD patients (*n* = 5).

For 12 patients (5 BMD and 7 DMD) we were able to obtain all three measurements for 4 years and follow the individual evolution of the biomarkers. Even if inter- and intra-individual variability of the miRNA levels is present, in both DMD and BMD patients, the general trend of decrease in the copy number of followed miRNAs is observed (Figure 4). We have performed repeated measurements analysis to test whether the behavior between the groups in the time is different or not.

**Figure 5:**
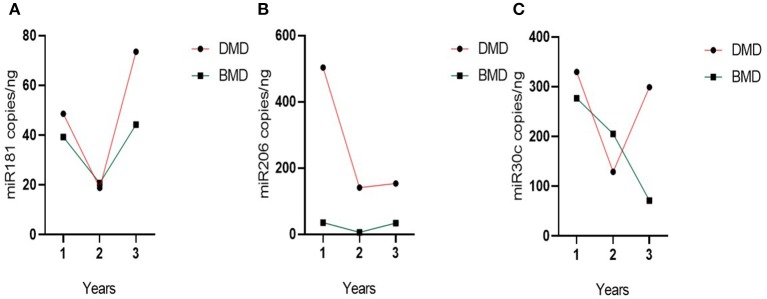
Evolution of group miRNAs levels in the time. **(A)** miR-181a, **(B)** miR-30c, and **(C)** miR-206. Evolution of the biomarker levels in the grouped DMD and BMD patients with three different time point measurements represented on the *X*-axis. Dots represent the mean of the group.

Evolution over time for miR-181a and miR-30c appears different in the DMD vs. the BMD group (Figure 5), but this difference does not reach statistical significance (*p* = 0.060 and 0.084, respectively). In contrast, in the case of miR-206 the two groups have a parallel behavior across time (Figure 5) and since miR-206 levels are much higher in DMD group than in Becker at all times, there is a significant difference between the groups globally over time (*p* = 0.001).

### Effects of Introducing the Corticosteroid Treatment on the miRNAs Levels

Six DMD patients from our cohort at the first time point measurements were steroid naïve at the start of the study and had been on corticosteroid regime which was introduced later. Four out of six patients were on the corticosteroid treatment for more than 6 months, one of them, <6 months and one patient for 6 months. We compared the levels of each miRNA before the treatment with the levels measured after the introduction of the corticosteroid treatment using the Wilcoxon test. We did not observe significant changes in the levels of the three miRNAs prior with respect to post-treatment in the six patients analyzed (data not shown).

### Analysis of miR-206 and miR30c Expression Levels in the Expanded Patient Cohort

Since miR30c and miR206 showed consistent differences with time in patients, we further validated the observations from the longitudinal study in an additional group of patients with wider age distribution and with different ambulatory statuses. For the new cohort of patients, the measurements of the miRNA levels were performed at the same time with the third time point measurements for the longitudinal cohort. Thus, the results presented here include the third time point measurement of the longitudinal cohort and the newly recruited patients.

MiR-206 expression levels showed significant differences in expanded DMD cohort (*n* = 25, mean = 160.6 copies/ng) in respect to controls (*n* = 13, mean value = 1.34 copies/ng). In the new BMD cohort (*n* = 15, mean value = 24.11 copies/ng) there is also significant difference in respect to controls (*p* = 0.0010).

MiR-30c expression levels in the expanded DMD cohort (*n* = 23, mean value = 250.0 copies/ng) are significantly different (*p* = 0.0283) from the controls (*n* = 13, mean value = 60.35 copies/ng). However, when the expanded cohort of the BMD patients (*n* = 14, mean value = 63.94 copies/ng) was analyzed there was no significant difference in respect to the controls.

No significant differences in the levels of both miR30c and miR206 are found when comparing very young DMD patients or not ambulant DMD patients with the rest of the DMD cohort.

### Correlation Between Age and Outcome Measures and Biomarkers

To determine if the observed differences between DMD and BMD patients and between the different time points and considering that DMD is a progressive degenerative disorder we investigated if circulating miRNA levels correlated with patient's age (grouping DMD and BMD together).

All three biomarkers showed a negative correlation with age, although the only biomarker close to being significant is miR206 in the first measurements (rho = −0.470, *p* = 0.077) and significant in the second time point (rho = −0.821, *p* < 0.001; Figure 3). However, in the third time point correlation with the expanded cohort of patients, the correlation is not significant (rho = −0.370, *p* = 0.0283).

To analyze whether miR-30c, miR-181a, and miR206 could be used as surrogate biomarkers of disease progression in ambulant DMD patients, we correlated expression levels in DMD and BMD patients with several validated functional outcome measures: NSAA, 6MWT, and TFTs which were recorded at the time when the blood samples were taken. No significant correlations have been found when we analyzed DMD and BMD cohorts separately and no significant differences have been found when correlation analysis has been performed with grouped DMD and BMD patients in the first time point measurement. In the second time point measurement, descend grade is significantly correlated with miR-181a and miR-30c (rho = 0.636 and 0.626, and *p* = 0.038 and 0.044, respectively) while miR-206 shows more strong significant negative correlation with 6MWT (rho = −0.759, *p* = 0,002).

In the third time point measurements there is a significant correlation of miR-206 and miR-30c with NSAA, climb time, descend time and grade, however, the correlation coefficients are week (data not shown).

### Analysis of miRNAs and Target Genes in Skeletal Muscle

**Figure 6:**
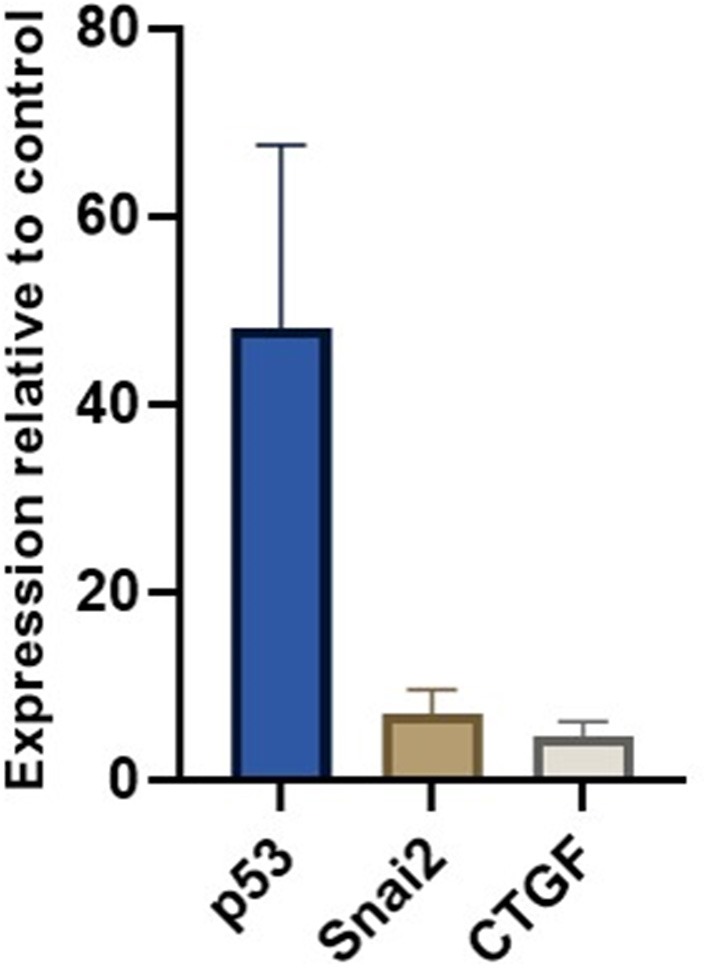
miR-30c target genes relative expressions in muscle. The borders of the columns represent mean values and error bars present SEM. Relative expression to controls and housekeeping genes measured in duplicates in three independent experiments.

We measured miR-30c, miR-181a, and miR-206 in skeletal muscle of DMD patients (*n* = 6) and found that miR-30c expression was decreased relative to the control muscle by 2-fold. Although this is a small change we hypothesized that as a result the expression of mRNAs downstream of miR-30c should be altered. Based on our previous results (19) and existing literature we chose p53, SNAi2 and CTGF as candidate genes since each of them is involved in an important aspect of DMD pathophysiology, namely, glucose metabolism, regeneration, and fibrosis. We found a marked up-regulation of all three genes in the muscle biopsies from those same DMD patients (fold change 40×, 7×, and 5×, respectively; Figure 6).

## Discussion

DMD is a lethal disease caused by the absence of dystrophin, which is still incurable. It leads to chronic muscle damage followed up by fibro-fatty substitution of muscle mass. The difference between DMD patients and healthy individuals is observed and described in changed gene expression, histological, and proteomic differences, enabling the identification of pathomechanisms and morphological alterations behind the clinical presentation. With intensive development of the advanced genetic therapies seen in the past few years, there is an increased and unmet need to monitor the effects of these therapeutic strategies in a more objective and quantitative manner. The antisense oligonucleotide-mediated exon skipping strategy aims to reverse the DMD phenotype to the less severe BMD phenotype (20). However, currently, there is no easy way to assess the switch from one phenotype to another in a quantitative manner and without invasive muscle biopsy, as for now, there is no consensus specific biomarker able to distinguish DMD from milder BMD patients. Altogether, this results in difficulties in determining patients benefit from the therapy. The intense quest for the specific biomarkers has resulted in several transcriptomic analyses being performed and potential miRNAs described to be good candidates. However, one needs to take into account the variety of biological processes in which miRNAs are implicated, when considering and proposing these molecules as specific biomarkers. For these reasons, we have decided to investigate the candidate miRNA signature in DMD and BMD patients, measured at different time points during 4 consecutive years.

Our results show that when challenged in the long-term study miR-181a fails to discriminate between the patient and the control population, possibly suggesting that this miRNA may be important in the earlier stages of the disease.

The myomiR, miRNA 206, is known to be elevated in the serum from patients with DMD and exhibits close to 100% specificity and sensitivity in distinguishing between DMD and healthy individual (8). It has been also reported that the levels of this myomiR in BMD patients are somewhere intermediary between DMD patients and controls. Our results strongly support these previous observations giving them further strength since we have validated this observation over time. This is important because despite its decrease with age miR-206 remains higher in patients. We were able to show that this miRNA is a good biomarker to distinguish between the DMD and BMD phenotypes regardless of the stage of the disease. We have not observed the significant difference in the levels of miR-206 in the expanded cohort of patients comparing not ambulant and ambulant patients or significantly higher levels of miR-206 in the very young DMD patients which are in contrast with the results of Zaharieva et al. but this differences could be attributed to the different techniques used (i.e., qPCR vs. absolute quantification with ddPCR) or differences in the patient cohorts. As mentioned earlier miR-206 is involved in the process of skeletal muscle injury and regeneration, and consequently, this miRNA has been found dysregulated in patients with other dystrophinopathies (13) and also in the mouse models for different muscular dystrophies (21). Thus, miR-206 may actually be very useful biomarker for dystrophic changes in the muscle. Certainly, further research on wider patient cohorts from different muscular dystrophies would point out if miR-206 levels could discriminate between different forms of muscular dystrophies, as we here notice the ability to discriminate DMD from BMD patients. Moreover, dysregulation of miR-206 has been described in mdx mouse model, following the same pattern, with higher expression on miR-206 observed in serum of the young mice and general decrease of the expression in the later assessment point of the 6 month old mice (21). The elegant study of Israeli et al. compared a palette of miRNAs in five mouse models of different muscular dystrophies. In the (KO-*Sgca*) mice model for α-sarcoglycanopathy and limb-girdle muscular dystrophy type 2DLGMD2D mice, the normalization of the miRNA expressions in serum are observed after injecting recombinant adeno-associated virus (rAAV9) expressing the human α-sarcoglycan cDNA (SGCA) gene, implying that miRNAs can serve as treatment efficiency biomarkers. Another study, in mdx mice has shown the dystromirs, including miR-206 responded to antisense oligonucleotide-mediated exon skipping therapy (22). Moreover, targeting miR-206 with AAV-mediated expression of a decoy target containing miR-206 (anti-miR-206), improves the muscle motor deficits in mdx mice and leads to overexpression of vascular endothelial growth factor A (VEGFA) and utrophin (23).

We also demonstrate the ability of the miR-30c to distinguish DMD population from healthy individuals at any time point and regardless on the stage of the disease. Our results show that this miRNA is not capable to separate BMD patients from healthy controls when a more diverse patient population was tested. This certainly does not exclude miR-30c as a good biomarker but calls for further analysis of the larger patient cohorts.

Our data, even limited with patient cohort size, in some manner represent the natural history of three miRNAs in DMD patients and as such could be of the great interest when the changes of miRNA expressions after molecular therapies as exome skipping, become available.

Taken together our results confirm the utility of the miR-206 and miR-30c as sensitive and specific biomarkers for DMD. Importantly, we demonstrate that miR-206 can successfully distinguish two phenotypic forms of the disease. We also show that it is important to verify in the long-term setup any candidate for the biomarker as we notice that all three miRNAs show the important extent of both inter and intrapatient variability which could also be a consequence of their involvement in the different biological processes taking places simultaneously.

## Data Availability Statement

The datasets generated for this study are available on request to the corresponding author.

## Ethics Statement

The studies involving human participants were reviewed and approved by Ethical Committee of Fundació Sant Joan de Déu. Written informed consent to participate in this study was provided by the participants' legal guardian/next of kin.

## Author Contributions

ST acquired and analyzed the data and drafted the manuscript. DN, JE, CO, LC, JM, CB, and AN collected and analyzed the clinical data, discussed the results, and participated in the final version. DC analyzed the data. CJ-M generated the conception of this work, analyzed and interpreted the data, and revised the manuscript. All authors read and approved the final manuscript.

## Conflict of Interest

The authors declare that the research was conducted in the absence of any commercial or financial relationships that could be construed as a potential conflict of interest.
